# Role for Sumoylation in Systemic Inflammation and Immune Homeostasis in *Drosophila* Larvae

**DOI:** 10.1371/journal.ppat.1001234

**Published:** 2010-12-23

**Authors:** Indira Paddibhatla, Mark J. Lee, Marta E. Kalamarz, Roberto Ferrarese, Shubha Govind

**Affiliations:** 1 Biology Department, The Graduate Center, The City College of the City University of New York, New York, New York, United States of America; 2 The Graduate Center, The City College of the City University of New York, New York, New York, United States of America; Stanford University, United States of America

## Abstract

To counter systemic risk of infection by parasitic wasps, *Drosophila* larvae activate humoral immunity in the fat body and mount a robust cellular response resulting in encapsulation of the wasp egg. Innate immune reactions are tightly regulated and are resolved within hours. To understand the mechanisms underlying activation and resolution of the egg encapsulation response and examine if failure of the latter develops into systemic inflammatory disease, we correlated parasitic wasp-induced changes in the *Drosophila* larva with systemic chronic conditions in sumoylation-deficient mutants. We have previously reported that loss of either Cactus, the *Drosophila* (IκB) protein or Ubc9, the SUMO-conjugating enzyme, leads to constitutive activation of the humoral and cellular pathways, hematopoietic overproliferation and tumorogenesis. Here we report that parasite infection simultaneously activates NF-κB-dependent transcription of *Spätzle processing enzyme* (*SPE*) and *cactus.* Endogenous Spätzle protein (the Toll ligand) is expressed in immune cells and excessive SPE or Spätzle is pro-inflammatory. Consistent with this function, loss of *Spz* suppresses *Ubc9^−^* defects. In contrast to the pro-inflammatory roles of SPE and Spätzle, Cactus and Ubc9 exert an anti-inflammatory effect. We show that Ubc9 maintains steady state levels of Cactus protein. In a series of immuno-genetic experiments, we demonstrate the existence of a robust bidirectional interaction between blood cells and the fat body and propose that wasp infection activates Toll signaling in both compartments via extracellular activation of Spätzle. Within each organ, the IκB/Ubc9-dependent inhibitory feedback resolves immune signaling and restores homeostasis. The loss of this feedback leads to chronic inflammation. Our studies not only provide an integrated framework for understanding the molecular basis of the evolutionary arms race between insect hosts and their parasites, but also offer insights into developing novel strategies for medical and agricultural pest control.

## Author Summary

Parasitoid wasps are a large group of insects in which the female injects her eggs into the bodies of host caterpillars (also called larvae). When the wasp egg hatches, the parasite larva gradually eats the host alive and takes over its body. Soon after the parasite egg is laid, an arms race between the parasite and the host is initiated. In a dramatic and highly restrained reaction, the host's blood cells surround and choke the development of the parasite egg. This encapsulation reaction allows the host to resume its development. We use *Drosophila* and its natural parasites to identify the mechanism that is essential for proper activation and termination of the encapsulation reaction. Unchecked encapsulation-like reaction flares up into a chronic inflammatory blood cancer in uninfected sumoylation-deficient larvae. Our studies reveal the parallels between acute (egg encapsulation) and chronic (blood cancer) inflammation in the fly. Moreover, these parallels match the criteria for acute and chronic inflammation in mammals. We can now understand more clearly how virus-like particles and factors introduced into the host along with the wasp egg disable the host's immune system to win the host/parasite arms race.

## Introduction

*Drosophila* are hosts to a range of pathogens, and in the wild, they likely encounter a large number of pathogen species [1], [2]. The survival of natural population structures of *Drosophila* spp. is expected to depend on the distribution of their pathogens, occurrence of co-infection, and specific host-pathogen interactions. One class of natural fly enemies is the parasitoid (parasitic) wasps, which, although free-living as adults, have an obligate relationship with their hosts for pre-imaginal development. Females inject 100 µm size eggs through the larval cuticle, directly into the host hemocoel, bypassing barrier tissues (cuticle, trachea, and gut). Because insects have an open circulatory system (there is no distinction between interstitial fluid and blood), the hemolymph bathes all internal organs in the hemocoel. Wasp egg recognition thus activates both systemic responses, blood cell proliferation and activation [3], [4], and the production of humoral factors from the fat body into the hemolymph [4], [5].

Blood cells recognize, surround, and melanize the parasite egg to sequester it from the host tissues. All three blood cells types, namely, the abundant phagocytic plasmatocytes, larger adhesive lamellocytes, and few melanin-producing crystal cells, are called into action [2], [3], [4], [5]. The encapsulation reaction is resolved within a day and is reminiscent of effector cell activation and resolution in mammals (e.g., tuberculosis granulomas [6]).

While cell-based immunity in *Drosophila* appears sufficient to restrain parasite development, it remains unclear why metazoan parasite infections trigger the Toll-dependent humoral arm in the fat body that is activated (and has been characterized) in response to microbial infections [2], [5]. Antimicrobial peptides, lysozymes and activation of the pro-phenoloxidase cascade (melanization) make up the humoral reactions. Both, the cellular and humoral immune reactions in healthy animals are regulated by conserved NF-κB, JAK-STAT and pro-phenoloxidase cascades [5], [7], [8]. Not surprisingly, many parasitic wasp species that infect *Drosophila* have evolved mechanisms to evade or suppress their host's immune responses [4], [5].

The goal of this study was to (1) identify the key mechanisms in *Drosophila* that contribute to both, the activation and resolution of parasitic wasp-induced acute inflammation, and (2) examine if the aberrant regulation of the latter sustains systemic chronic inflammation in hosts. We were guided by the paradigm of acute inflammation in mammals, which is characterized by proliferation, differentiation, and recruitment of blood cells to the site of injury. Acute-phase gene expression is self-limiting, being quickly resolved within minutes to hours after infection [9], [10]. Innate effector cells aggregate to sequester the invading parasite or microbe. Examples of such reactions include granulomas around parasitic helminthes, which involve neutrophils, macrophages or T cells [11] and inflammatory granulomas of tuberculosis-inducing *Mycobacterium tuberculosis* by foamy macrophages [6]. Inflammatory cells produce pro-inflammatory cytokines (TNF-α and interleukins), chemokines, and prostaglandins, which help initiate and mediate the inflammatory processes by activation of NF-κB signaling [9], [10].

The aberrant regulation of this inflammatory response in humans has been identified as a common denominator underlying many conditions such as cancer, diabetes, and heart disease. Chronic inflammation is caused by, among others, the continuous activation of acute inflammatory pathways [12]. At times, acute inflammation can spiral out of control and cause major organ failure and death.

To understand how activation and resolution of acute inflammation are coordinated in the fly, we examined patterns of expression in genome-wide microarray data of *Drosophila* infected by distantly-related parasitic wasps (*Leptopilina boulardi* [5] and *Asobara tabida* [8]). We found that the transcription of two core components of the Toll pathway, *Spätzle Processing Enzyme* (*SPE*) and *cactus* (*cact*), is strongly activated in the first six hours after infection. The SPE protease is constitutively expressed in the larval fat body and blood cells. It cleaves and activates the Toll ligand pro-Spätzle (encoded by *spz*) [13], [14]. Spz, is a cysteine-knot protein; the *spz* locus itself encodes multiple alternatively-spliced isoforms [15]. *SPE* transcription is induced by injury via a Toll-dependent positive feedback loop. Loss or depletion of SPE impairs the induction of *Drosomycin* (*Drs*), a target gene of the Toll pathway [13], [14].

Loss of function of the fly IκB, *cact*, [16] or the SUMO conjugase, *Ubc9* (also called *lesswright*, *lwr* [17], [18]), results in hyperactive Toll/NF-κB signaling. Mutant larvae show constitutive activation of humoral (antimicrobial and immune peptide gene expression in the fat body, [19]) and cellular (blood cell proliferation, aggregation and microtumor formation) reactions [17], [18]. Transcriptional activation of *cactus* after bacterial challenge, like that of *SPE*, is under the control of the Toll-Dorsal pathway. High levels of Cactus protein terminate signaling [20]. Several components in the mammalian NF-κB pathway, including IκBα, are regulated by sumoylation. The SUMO (small ubiquitin-like modifier) modification system utilizes SUMO-activating and -conjugating enzymes, Uba2/Aos1 and Ubc9, respectively [21]. Sumoylation plays a significant role in host defense in plants, insects and mammals [22], [23].

With this backdrop, we hypothesized that the encapsulation of the wasp egg is akin to the mammalian acute inflammatory reaction, whereas hyperactive Toll signaling in *Ubc9^−^* mutants is a deregulated chronic condition. We sought to systematically define this parallel in the fly larva and discovered the central role of Ubc9 in the regulation of the core, primordial NF-κB immuno-genetic circuit, where it governs the balance between pro-inflammatory (SPE/Spz) and anti-inflammatory (Cactus) molecules. Our findings provide a molecular model for understanding the immune physiology underlying the evolutionary arms race between insect hosts and their parasitic wasps.

## Results

### Sumoylation restrains systemic inflammation

**Figure 1:**
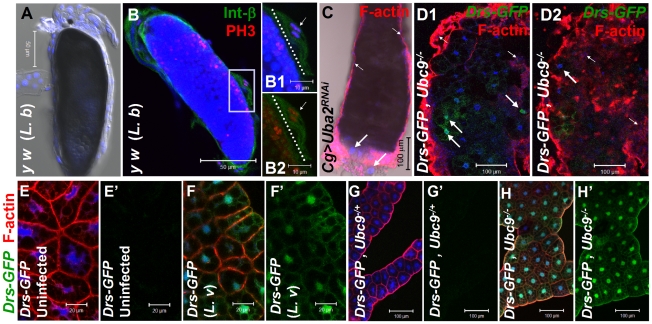
Inflammatory responses in the fly larvae. (A) Melanized wasp (*L. boulardi*) egg encapsulated by blood cells. (B) Integrin β PS (green) is expressed in most blood cells of the capsule. Some blood cells are mitotically active, where the phospho-histone H3 (PH 3, red) signal overlaps with DNA (B1, B2). Inset shows higher magnification of wasp embryo to which integrin β PS-positive (blood cells, [52]) and some PH 3-positive blood cells adhere (short arrows in B1–B2). PH 3-positive cells are also detected within the developing wasp embryo. Broken line shows the wasp embryo/host capsule interface (B1, B2). (C) Melanized microtumor showing fat body encapsulated by blood cells in *Cg>Uba2^RNAi^* larva. Microtumors are structures larger than aggregates of more than 50 blood cells, but smaller than 1 mm^3^ in volume. Long arrows point to fat body and short arrows to blood cells adhering to the fat body. Blood cells are diploid and significantly smaller than the larger, endopolyploid fat body cells. (D1, D2) Confocal sections of a microtumor (D1, tumor interior; D2 tumor periphery) showing blood cells (short arrows) infiltrating fat body cells. Some fat body cells show constitutive *Drs-GFP* expression (long arrows). (E–H) *Drs-GFP* expression in fat body before (E, E'), or after (F, F') wasp (*L. victoriae*) infection. (G–H) *Drs-GFP* expression in heterozygous (G, G'), and *Ubc9^−^* (H, H') fat body. Panels E', F', G' and H' show *Drs-GFP* transgene expression alone. (A–H) Cells counterstained for DNA with Hoechst (blue).

There are striking parallels between wasp-induced activation of cellular and humoral immunity in control larvae and hyperactive immune phenotypes of *Ubc9^−^* larvae. First, parasite infection induces some blood cells to divide, differentiate, and aggregate. Blood cells are recruited to surround the melanized wasp egg to form the capsule structure itself (Figure 1A, B–B2). Loss of sumoylation via RNA interference in blood cells and fat body (*Cg>Uba2^RNAi^* Figure 1C; [24]), or in *Ubc9^−^* mutants (Figure 1D1, D2) leads to similar changes. Second, while *Drs-GFP*, a transgenic Toll pathway readout is normally induced only after parasite infection of control larvae (Figure 1E–F'), *Ubc9^−^* fat body cells constitutively express this reporter (Figure 1 G–H').

**Figure 2:**
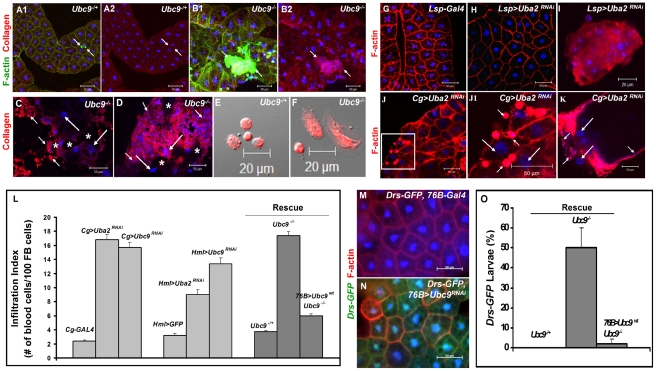
Loss of sumoylation enzymes induces loss of fat body integrity, hematopoietic defects, infiltration, and *Drs* activation. (A–B) Merged Z-stack sections of 6-day-old heterozygous (A1, A2), or *Ubc9^−^* (B1, B2) fat body stained for Collagen IV. Short arrows point to single blood cells or clusters of blood cells in contact with the fat body tissue. Panels A2 and B2 show the anti-Collagen IV antibody signal only. (C–D) Anti-Collagen IV antibody staining of 8-day-old mutant fat body (C) or microtumors (D). Regions of the fat body with no Collagen IV signal are marked with an asterisk. Blood cells (short arrows) infiltrating the fat body (long arrows). (E–F) Blood cells from control and mutant larvae stained for Collagen IV. (G–K) Fat body from control *Lsp-Gal4* (G) and *Lsp>Uba2^RNAi^* (H). A hematopoietic aggregate from *Lsp>Uba2^RNAi^* hemolymph (I). Fat body from *Cg>Uba2^RNAi^* larva (J, J1). Inset in panel J (white square) is shown at high magnification in panel J1. Microtumor from a *Cg>Uba2^RNAi^* larva (K). Blood cells (short arrows) encapsulating fat body (long arrows) in *Cg>Uba2^RNAi^* larva (J1, K). (L) Infiltration indices in third instar larvae (see Methods). *n*>20 larvae for all genotypes except *Hml>Uba2^RNAi^*, *Hml>Ubc9^RNAi^*, and *Cg>Ubc9^RNAi^*, where *n*  = 8 larvae. Bars show standard error. (M–N) No *Drs-GFP* expression in control (*76B-Gal4*) larval fat body (M). *Drs-GFP* expression is induced in the experimental (*76B>Ubc9^RNAi^*) fat body (N). (O) Rescue of constitutive *Drs-GFP* expression in *Ubc9^−^* mutants with *76B>Ubc9^WT^* transgenes *n*>90 animals. Cells in A–B, E–K and M–N are counterstained for DNA with Hoechst (blue).

We hypothesized that the fat body infiltration by blood cells in sumoylation-deficient *Cg*>*Uba2^RNAi^* or *Ubc9^−^* animals represents a chronic version of the egg recognition/encapsulation reaction. To understand the nature of the blood cell-fat body interaction, we stained cells of 6-day-old control and *Ubc9^−^* mutants with anti-Collagen IV antibody [25]. Collagen IV, a component of the basement membrane, is expressed uniformly around the cells of control fat body (Figure 2A1, A2). However, the staining signal for Collagen IV around the fat body of 6-day (Figure 2B1, B2) and 8-day-old (Figure 2C) mutant larvae is discontinuous. In 6-day-old samples, the Collagen IV-deficient regions coincide with low or undetectable F-actin signal, suggesting loss of tissue integrity. Aggregates of blood cells around such collagen-deficient regions are frequently observed in 8-day-old fat body (Figure 2C, D). These results suggest that mutant blood cells are actively recruited to regions of the fat body with irregular basement membrane, in a process that is likely to be similar to infiltration and aggregation of blood cells around the wasp egg. The Collagen IV staining signal in control and mutant blood cells is high and primarily cytoplasmic (Figure 2E, F), consistent with the high promoter activity in the *Cg-Gal4* strain.

To examine the relative contributions of fat body and blood cells to blood cell activation and infiltration, we compared the effects of sumoylation knockdown (*Uba2^RNAi^*) in fat body (*Lsp-Gal4*), blood cells (*Hml-Gal4*), or both these tissues (*Cg-Gal4*). Whereas knockdown of Uba2 in the larval fat body alone did not yield strong fat body defects (i.e., loss of tissue integrity, Figure 2G, H), it resulted in weak but reproducible differentiation and aggregation of blood cells in the hemocoel (Figure 2I). In contrast, *Cg>Uba2^RNAi^*, *Cg>Ubc9^RNAi^*, *Hml>Uba2^RNAi^* or *Hml>Ubc9^RNAi^* larvae mimic defects of *Ubc9* mutants, significantly promoting infiltration (number of single hemocytes per 100 fat body cells; Figure 2J, J1, L) and tumorogenesis (Figure 1C and Figure 2K). Whereas 60% of the *Cg>Uba2^RNAi^* or *Cg>Ubc9^RNAi^* animals developed microtumors, none of the *Cg-Gal*, *UAS-Uba2^RNAi^*, or *UAS-Ubc9^RNAi^* parents were tumorous in third-instar larval stages (n>30 larvae for all genotypes; data not shown).

To check if sumoylation-deficient blood cells can signal the wild type fat body to activate *Drs-GFP*, we examined the *76B>Ubc9^RNAi^* animals and found that deficiency in even a few blood cells of the lymph gland is sufficient to trigger *Drs-GFP* activation (Figure 2M, N). The ability of blood cells to activate *Drs-GFP* in wild type fat body was confirmed in *76B>Aos1^RNAi^* animals (data not shown). (The *76B* driver is expressed in few cells of the lymph gland [26], the multi-lobed larval hematopoietic organ [2]. Aos1 is a SUMO-activating enzyme subunit.).

These results suggest that (1) the sumoylation of target proteins in the fat body inhibits the release of pro-inflammatory factors, and (2) blood cells lacking this protein modification pathway become inflammatory, *i.e*., they divide, differentiate, release pro-inflammatory signals and infiltrate the fat body in the absence of infection. Loss of sumoylation in both immune tissues amplifies these defects.

If deficiency of sumoylation in blood cells of *Ubc9^−^* mutants or *Aos1/Ubc9* knockdown animals elicits factors that can activate pathways in the fat body, then supplying wild type Ubc9 protein in blood cells should, to a large extent, restore immune homeostasis in both tissues. We tested this idea in a rescue experiment in which wild type Ubc9 protein was expressed only in a subset of the lymph gland cells (*76B>Ubc9^WT^*) in *Ubc9^−^* mutants. Not only was the infiltration index significantly reduced in *76B>Ubc9^WT^* mutants (Figure 2L), but surprisingly, the *Drs-GFP* reporter in the fat body was no longer expressed [i.e., while 50.3±9.6% of mutants (n>35 animals) show *Drs-GFP* expression, only 2.1±2.1% of mutants expressing *Ubc9^WT^* transgene (n>35 animals) are *Drs-GFP*-positive, Figure 2O].

All together, these results support the idea that Ubc9 plays an anti-inflammatory role in both, the fat body and blood cells. Loss of Ubc9-dependent inhibition leads to failure of immune homeostasis and the development of chronic inflammation. This interpretation implies that each immune tissue can activate the other by secretion of cell non-autonomous factors and thus, mutually alter the patterns of gene expression and cell decision processes.

### Identification of pro- and anti-inflammatory factors

To clarify the underlying molecular parallels between infection and the genetic loss of sumoylation, we utilized microarray datasets to examine patterns of gene expression in parasite-infected fly larvae and identified genes with acute-phase expression profile, typical of mammalian inflammation [12]. In hosts infected by *A. tabida* and *L. boulardi* [5], [8], we identified 81 genes with acute-phase expression pattern (Figure S1A, B; see Table S1 for the identity of genes). Forty genes were activated after *A. tabida* infection (Wertheim study [8]), and 51 genes were induced by *L. boulardi* infection (Schlenke study [5]). Ten genes were induced by infection with either wasp, and thus, identified in data sets from both studies.

A majority of the 81 genes are linked to the immune response of the fly (*Irc* and *Idgf* family members) and roughly a quarter of genes encode components or targets of the Toll (*PGRP-SD, SPE, nec, Mtk, Tl, cactus, IM2, IM3, IM23*), Imd (*PGRP-LB, Relish, AttA, B, mtk), JAK-STAT* (*dome, hop*) or melanization (*yellow-f, Dox-A3*) pathways (Table S1). A handful of genes, not implicated in immune, development, metabolism, or other processes (e.g., *Ugt86Dd, CG32687, CG7896, CG9095*) were also identified (Table S1). Significantly however, the 81 putative acute inflammation genes include components that act upstream (*PGRP-SD*, *SPE* and *Tl*) and downstream (*cact*) of Ubc9 itself. Since the expression of *SPE* and *cactus* was previously shown to be regulated by Toll-dependent feedback [13], [14], [20], we hypothesized that their coordinate regulation is essential for both activation and termination of acute inflammation.

### The levels of *SPE* and *cactus* depend on NF-κB proteins Dorsal and Dorsal-related immunity factor

**Figure 3:**
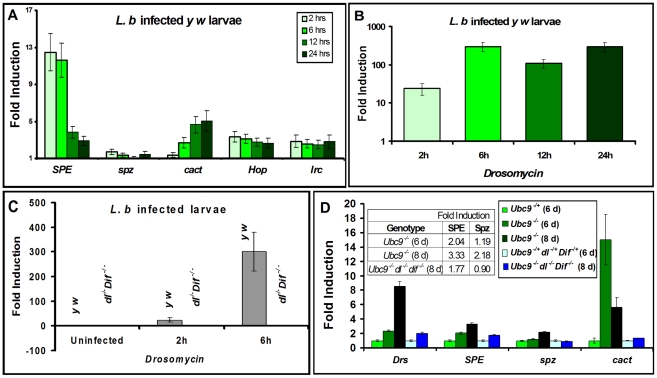
Inflammatory responses are dependent on Dorsal/Dif. (A–D) Real-time PCR on RNA from whole larvae. (A) Time course of post infection (*L. boulardi*) of *SPE*, *spz, cact, hopscotch* and *immune-regulated catalase* activation relative to uninfected wild type (*y w*) animals. (B) *Drs* activation at different time points after *L. boulardi* infection in wild type *y w* animals. (C) *Drs* activation at 2 h or 6 h after *L. boulardi* infection in wild type (*y w*) or *Dif^−^ dl^−^* animals. (D) Gene expression in single or triple mutants compared to respective heterozygotes. Table (D, inset) shows numeric fold increase for *SPE* and *spz* in single and triple mutants. Bars represent standard errors for results from triplicate measurements.

We quantified *SPE*, *spz*, *cactus* and *Drs* transcription in parasite-infected (Figure 3A, B) and mutant animals (Figure 3C, D). Parasite infection activates *SPE* 10-fold higher than uninfected controls in the first six hours of infection, subsequent to which, its expression is reduced as RNA levels return below 5-fold at 12 and 24 hours. Unlike *SPE*, *spz* RNA levels remain low. However, RNA levels of *cact* increase significantly (up to 5-fold) over 24 hours post-infection. Transcription of two other immune genes (not core components of the Toll pathway), *hop* and *Irc*, is activated (2-3-fold), but RNA levels remain relatively constant at all time points tested (Figure 3A).

Toll pathway activation was confirmed by quantifying expression of *Drs*. Parasite infection of control *y w* larvae activates *Drs* more than 100-fold (Figure 3B, C) and its constitutive expression in *Ubc9^−^* mutants is roughly 8-fold higher relative to heterozygous controls (Figure 3D). Like *Drs*, both *SPE* (2 fold) and *cact* (15 fold), but not *spz*, are constitutively expressed in 6-day old *Ubc9^−^* mutants (Figure 3D). Eight-day-old mutants exhibit stronger cellular immune defects [17], and RNA levels of *Drs* (8 fold), *SPE* (3 fold), and *spz* (2 fold) are higher relative to their 6-day-old counterparts (Figure 3D). This observation suggests a progressive loss of gene regulation in older animals.

To formally test the dependence of both acute (parasite-induced Toll activation in wild type) and chronic (constitutively-active Toll signaling in *Ubc9^−^* mutants) inflammatory responses on the transcription factors Dorsal (dl) and Dorsal-related immunity factor (Dif), we quantified *Drs* expression in animals lacking *Dif* and *dl* (Figure 3C, D). Under both conditions of infection (Figure 3C) and mutation (Figure 3D), *Drs* expression is almost completely dependent on these NF-κB proteins.

Like *Drs*, the constitutive expression of *cact* (at 6 day), *SPE* (at 6 and 8 day) and *spz* (at 8 day) in *Ubc9^−^* animals also depends on Dorsal/Dif as their normal transcription is completely or partially restored in *Ubc9^−^ Dif^−^ dl^−^* triple mutants (Figure 3D). Consistent with these findings, high confidence Dorsal-binding sites are present in genomic regions of *SPE* and *cact* ([20]; Figure S2).

**Figure 4:**
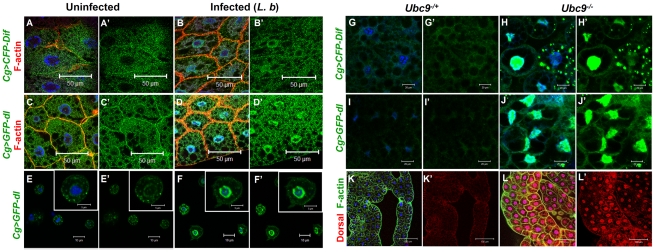
Nuclear localization of Dorsal and Dif in inflammation. (A–D) Fat body cells from uninfected *y w*; *Cg>CFP-Dif* and *yw*; *Cg>GFP-dl* larvae (A, A', C, C') show abundant vesicular localization of fusion proteins in the cytoplasm. After infection, these proteins are also nuclear (B, B', D, D'). (E–F') Cytoplasm-to-nuclear relocalization of GFP-Dorsal in blood cells after infection. (G–J) CFP-Dif and GFP-Dorsal in heterozygous (G, G', I, I') and *Ubc9^−^* (H, H', J, J') fat body cells. (K, L) Endogenous Dorsal protein expression (red) in heterozygous (K, K') and *Ubc9^−^* (L, L') fat body. Cells in panels A–L are counterstained with Hoechst to visualize nuclei (blue). Panels A'–J' show the green channel only. Panels K' and L' show the expression of Dorsal alone.

Parasite infection promotes the nuclear localization of both NF-κB proteins Dif and Dorsal in wild type fat body (*Cg>CFP-Dif, Cg>GFP-Dorsal*; Figure 4A–D) and blood cells (Figure 4E–F). Constitutive nuclear localization of both fusion proteins is observed in *Ubc9^−^* animals (Figure 4G–J). Nuclear localization of endogenous Dorsal was confirmed by antibody staining. Mutant fat body cells have higher Dorsal protein levels (compare Figure 4K' with L'), an observation that is in agreement with higher steady state *dl* RNA levels in *Ubc9^−^* mutants relative to heterozygotes (data not shown). Finally, a higher proportion (> 40%) of mutant blood cells exhibit nuclear Dorsal protein relative to heterozygous (<20%) controls (our unpublished results and [18]).

The parallels (with respect to NF-κB-dependent gene expression changes and hematopoietic activation, infiltration and associated changes) and differences (in regulation) defined in the preceding experiments in infected and mutant larvae support the notion that sumoylation of specific proteins coordinates the activation and deactivation of canonical Toll signaling in fat body and blood cells in part by regulating the steady state levels of SPE and Cactus. Furthermore, the *Ubc9^−^* animals suffer from chronic inflammatory phenotypes because of their inability to properly terminate Toll signaling.

### SPE and Spätzle are proinflammatory

**Figure 5:**
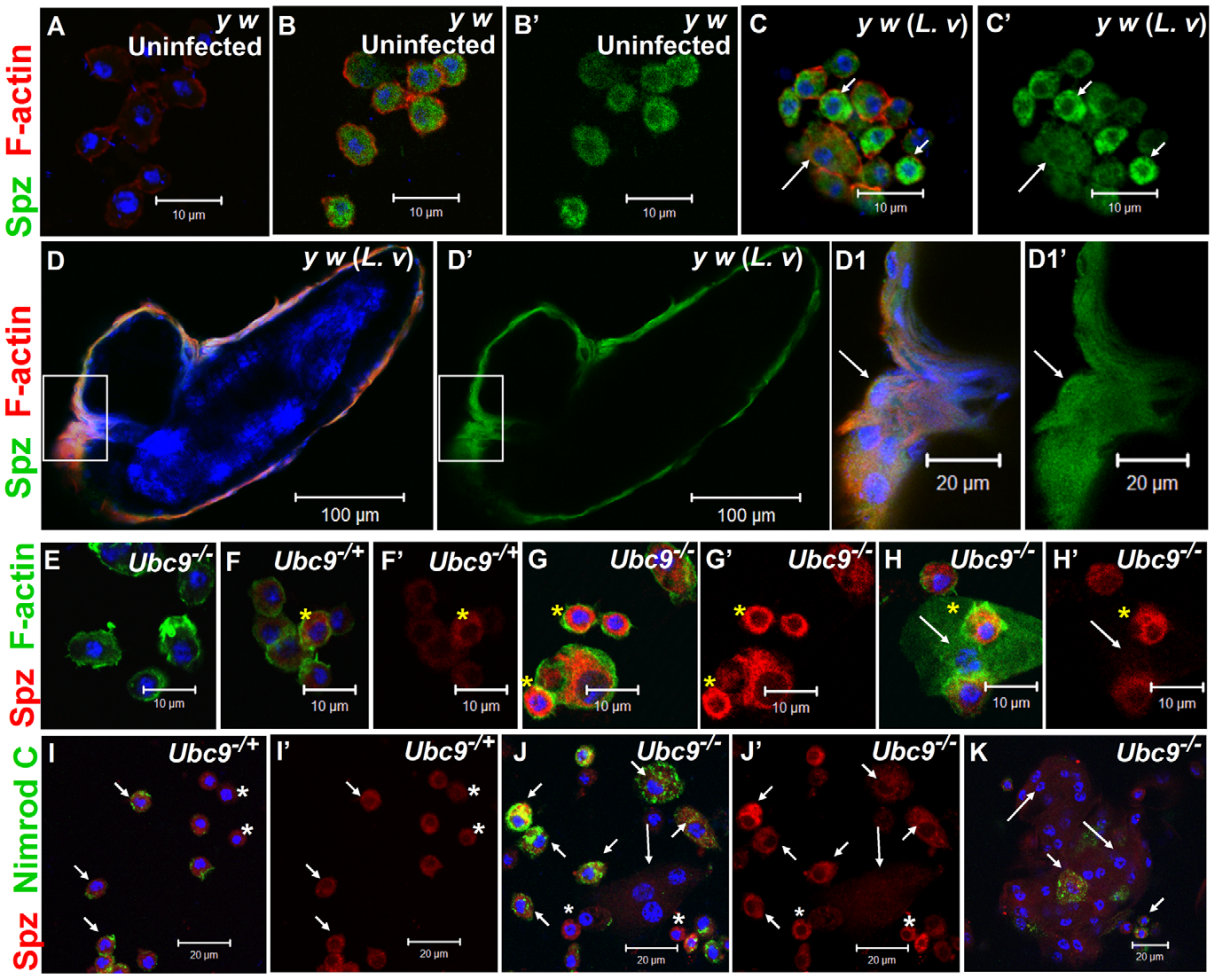
Spätzle expression in larval blood cells. (A–C') Infection: Blood cells from control larvae not treated with primary antibody (A). Spätzle expression (green) in blood cells from uninfected *y w* (B, B') and *L. victoriae*-infected *y w* (C, C') larvae. Lamellocytes (long arrow) in panel C show low but detectable Spz expression that is higher compared to plasmatocytes (short arrows). (D–D1') Spz expression (green) in blood cells surrounding the wasp (*L. victoriae*) egg (D). At high magnification (D1, D1'), Spz expression (green) in lamellocytes (arrow) colocalizes with F-actin (red). (E–H') Mutants: *Ubc9^−^* blood cells not treated with primary antibody (E). Spz signal (red) in heterozygous cells (F, F') is lower than in mutant (G, G', H, H') cells. Spz is less abundant in lamellocytes (H, H' arrow) than plasmatocytes. Asterisk denotes cells in which there is partial colocalization of the Spz (red) and F-actin (green) signals. (I–K) Spz (red) colocalizes with Nimrod C (green) in *Ubc9^−/+^* plasmatocytes (short arrows, I) and *Ubc9^−^* cells in circulation (J) or in a small aggregate (K). Small round blood cells (I, I', J, J'; asterisk) are Nimrod-negative, but express Spz. Long arrows point to lamellocytes. (A–K) Cells counterstained for DNA with Hoechst (blue). All the panels with a prime letter show the expression of Spz alone.

To understand its role in systemic inflammation, we next examined the expression of Spätzle in larval blood cells and fat body. Polyclonal anti-Spätzle antibodies [14] reveal Spz expression in the cytoplasm of control (*y w* or *spz^−/+^*), but not in *spz^−/−^* blood cells (Figure 5A, B; Figure S3A–C). Spz protein level is high after parasite infection of *y w* animals, particularly in circulating plasmatocytes (Figure 5B, B', C, C'). Spz protein is also clearly detected in plasmatocytes and lamellocytes layered around the wasp egg (Figure 5D–D1').

**Figure 6:**
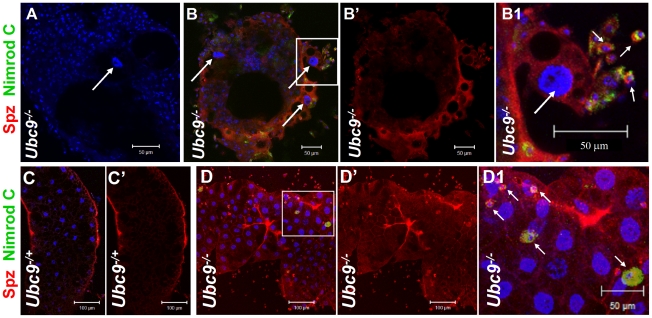
Spätzle protein expression in immune tissues of *Ubc9* mutants. (A–B1) *Ubc9^−^* samples. Primary antibody omitted (A). Plasmatocytes (Nimrod C, green, short arrows) around fat body cell (long arrow) express high levels of Spz (red). Inset in panel B is shown at high magnification in panel B1. (C–D1) Heterozygous (C, C') or *Ubc9^−^* (D, D') fat body stained with anti-Spz antibody. Infiltrating mutant plasmatocytes (D1, short arrows) express high levels of Spz. Panels B', C' and D' show the red channel alone. (A–D) All cells counterstained for DNA with Hoechst (blue).

Spz levels are high in uninfected *Ubc9^−^* blood cells (Figure 5G, G', H, H', J, J', K) compared to heterozygotes (5F, F', I, I'). In some cells, the Spz signal overlaps with F-actin (Figure 5F–H, asterisk) or Nimrod C (plasmatocyte marker) in circulating (Figure 5I–K), tumorous (Figure 6B, B, B1', short arrows), or fat body-infiltrating plasmatocytes (Figure 6D, D1, short arrows). Spz protein levels are higher in fat body of infected animals (data not shown) and mutant fat body cells relative to uninfected heterozygous cells (Figure 6C, C, D, D'' and Figure S3D–F'). High levels of Spz protein in cells of infected animals (or in mutant blood cells engaged in infiltration of mutant fat body) suggest that Spz serves a novel pro-inflammatory role in the activation of parasite-induced immune responses.

**Figure 7:**
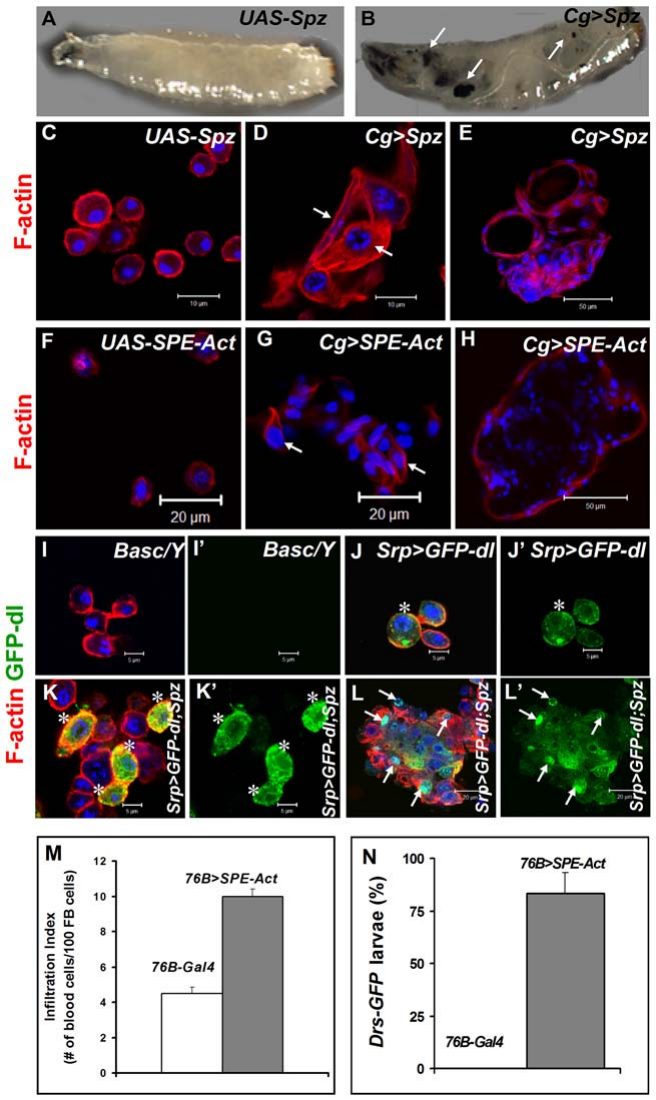
Excessive SPE, Spz and Dorsal proteins activate inflammation in the larva. (A, B) Misexpression of Spz induces blood cell aggregation and melanization. Control third instar larva with only the *UAS*-*Spz* transgene (A). Experimental *Cg>Spz* larva with excessive Spz in blood cells and fat body (B). Arrows point to melanized microtumors visible through the transparent cuticle. (C–H) Either Spz (D, E) or activated SPE (G, H) expressed via Gal4 drivers as shown. Wild type plasmatocytes in parental (*UAS-Spz*; C and *UAS-SPE-Act*; F) control animals. Arrows point to blood cells of lamellocyte morphology within a hematopoietic aggregate (D, G). Small microtumors from experimental animals (E, H). (I–L) High levels of Spz synergize with GFP-Dorsal to promote localization of the latter to the nucleus. Blood cells from control (*Basc/Y*, I, I') larva, without any transgene, lack GFP expression. GFP-Dorsal in blood cells from *Srp>GFP-dl* (J, J') larva is predominantly cytoplasmic (asterisk). Addition of excessive Spätzle (*Srp>GFP-dl*; *Spz*, K, K', L, L') promotes blood cell aggregation. GFP-Dorsal levels are high and the protein is both cytoplasmic and nuclear (K, K' asterisk) or predominantly nuclear (L, L' arrows). (C–L) Cells were counterstained with Hoechst (blue). Panels I', J', K' and L' show the expression of GFP-Dorsal alone. (M) Infiltration index in third instar larvae. Genotypes are shown. Bars show standard error. (N) *Drs-GFP* expression in *76-Gal4* (control) and *76B>SPE-Act* animals (n>45).

We next tested the effects of ectopic expression of full-length Spz and activated SPE (*SPE-Act*) with various drivers. Experimental animals with excessive Spz or SPE-Act either in the fat body (*Lsp-Gal4*, [27]) or hematopoietic (*Hml-Gal4,* [28]) or both (*Cg-Gal4*, [24]) compartments exhibit variable hematopoietic defects similar to those in *Ubc9^−^* and *cact^−^* third instar larvae (Figure 7A–H, [16], [17]). A similar outcome is observed upon over-expression of Dorsal or Dif ([18], [29], our unpublished results).

To determine if ectopic Spz can promote the nuclear localization of Dorsal, we monitored the subcellular distribution of GFP-Dorsal expressed in all blood cells (*Srp>GFP-dl*) with added Spz (*UAS-Spz*). In the absence of ectopic Spz, GFP-Dorsal is predominantly cytoplasmic in circulating blood cells (Figure 7J). Additional Spz expression promotes lamellocyte differentiation and some, but not all, blood cells show high levels of nuclear GFP-Dorsal, especially in microtumors (Figure 7K–L', arrows). Together, these observations suggest that Spz is a proinflammatory cytokine and its regulated activation after infection is an important step for the onset and resolution of the immune responses in *Drosophila*.

To examine if SPE derived from blood cells is sufficient to activate them and induce *Drs-GFP* in the fat body, we examined *76B>SPE-Act* carrying the *Drs-GFP*. Infiltration index in experimental (*76B>SPE-Act*) animals was significantly higher than in control (*76B-Gal4*) animals (Figure 7M). Furthermore, while the transgene remains inactive in the fat body of *76B-Gal4* control animals at both second and third instar stages, the experimental *76B>SPE-Act* larvae strongly express the *Drs-GFP* transgene at both stages (Figure 7N).

### Loss of *SPE/spz* suppress systemic inflammation

**Figure 8:**
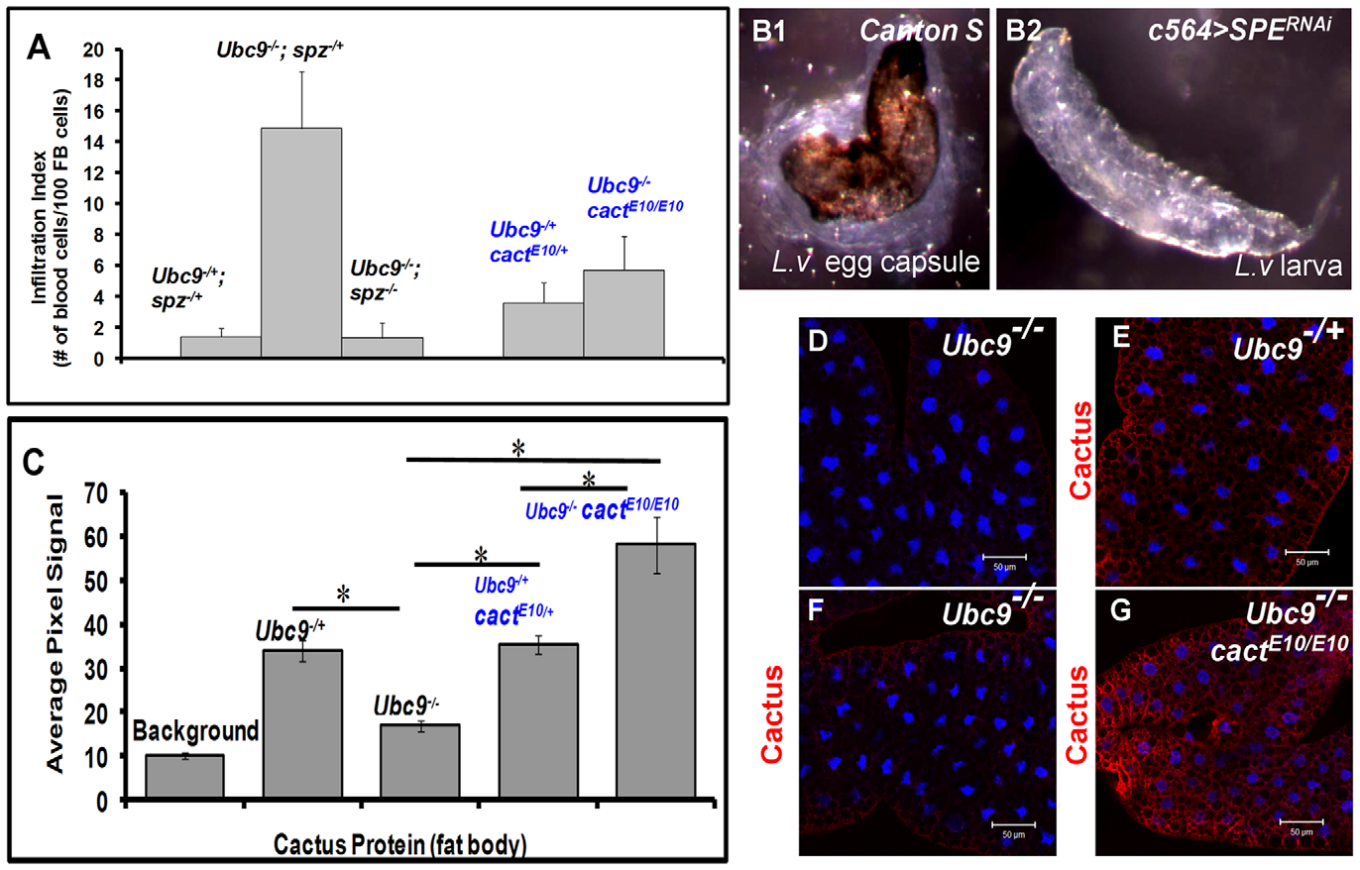
Inflammatory phenotypes depend on levels of Spz/SPE and Cactus. (A) Infiltration index in different genetic backgrounds. For all genotypes, *n* = 20, except for double mutants (*Ubc9^−/−^; spz^−/−^)*, where *n* = 5 animals. Bars show standard error. (B1, B2) Outcome of *L victoriae* infection in *Canton S* (control) and *c564>SPE^RNAi^* hosts. (C) Pixel signal quantification (see Methods) of Cactus protein levels in larval fat body. Genotypes are shown. Bars show standard error. Asterisk indicates significant difference (p<0.05). (D–G) Sample in panel D was not treated with anti-Cactus antibody (background). Cactus levels in fat body cells of *Ubc9^−/+^* (E), *Ubc9^−/−^* (F), or *Ubc9^−/−^ cact^E10/E10^* (G) animals. (D–G) Cells were counterstained with Hoechst (blue).

The preceding results, *i.e.,* (1) acute-phase activation of *SPE* after parasite infection (Figure 3A); (2) its elevated expression in *Ubc9^−^* mutants (Figure 3D); (3) high levels of Spätzle (Figure 5, 6) and SPE [13], [14] in immune cells; and (4) systemic inflammatory effects of the misexpression of either protein (Figure 7) suggest that a positive feedback loop through SPE/Spz supports the activation and resolution of infiltration, encapsulation and tumorogenesis. Consistent with this idea, we found that loss of *spz* function relieves the chronic effects of the *Ubc9* mutation (Figure 8A). Wandering third-instar *Ubc9^−^; spz^−^* double mutant larvae are almost free of microtumors and show a significant reduction in fat body infiltration index (number of single hemocytes/100 fat body cells, Figure 8A).

To test the specific contribution of SPE for normal encapsulation of the wasp egg, we infected *c564>SPE^RNAi^* animals (*c564-Gal4* is expressed in larval fat body and blood cells [30]). While experimental animals were completely immune compromised (0/153 infected animals showed encapsulation), control *Canton S* larvae efficiently (83.3%; n = 120 animals) encapsulated *L. victoriae* eggs (Figure 8B1, B2). These results strongly support the notion that high levels of SPE present in the hemolymph after infection are essential for encapsulation.

### Ubc9 controls stability of Cactus protein

To further examine how transcriptional and translational regulation of *cactus* modulates systemic inflammation and delineate the function of Ubc9 in this process, we first confirmed the transcriptional activation of the *cact^255^-lacZ* allele [31] in immune cells after parasite infection. Consistent with the temporal profile observed in whole larvae (Figure 3A), the fat body (Figure S4A–C) and blood cells (plasmatocytes, Figure S4D, F, H; lamellocytes; Figure S4G, G') of *cactus^255^/+* larvae showed significant activation of *cact* transcription. Consistent with this regulation, fat body and blood cells from parasitized *y w* animals showed increased level of Cactus protein (Figure S4I–L and data not shown). Thus, Cactus-dependent feedback is important for the resolution of wasp-induced acute inflammation.

In contrast to increased Cactus mRNA and protein after wasp infection, we were surprised to find that although *cact* RNA is higher in *Ubc9^−^* mutants relative to their heterozygous siblings (Figure 3A and S4), Cactus protein levels in mutant fat body are roughly half of that found in heterozygous fat body (Figure 8C–F), although this reduction is not as pronounced in the mutant blood cells (data not shown). Additionally, we examined Cactus levels in *Ubc9^−^ cact^E10^* double mutant fat body. This *cact* allele encodes a degradation-insensitive form of Cactus [32]. We found that, whereas single or double heterozygotes show roughly equal levels of Cactus (twice of that in *Ubc9^−^* fat body), the levels in *Ubc9^−/−^ cact^E10/E10^* fat body are roughly twice in comparison to controls (Figure 8C, G).

These results suggest that systemic chronic inflammation in *Ubc9^−^* larvae is, in part, sustained by reduced stability of endogenous Cactus in fat body cells. Significantly, the stable form of Cactus^E10^ protein suppresses blood cell infiltration, tumorogenesis, and expression of *Drs-GFP* in *Ubc9^−^* mutants (Figure 8A, C).

## Discussion

### Coordination and calibration of immune responses

Parasitic wasps are a large group of insects that typically attack other insects. Because of the absolute dependence on their insect hosts, parasitic wasps are of enormous commercial interest and can replace insecticides to control insect pests. The motivation of this study was to gain a clearer understanding of how insect larvae respond to attacks of these natural enemies. Using an immuno-genetic approach in *Drosophila*, we found that the same Toll-dependent NF-κB mechanism that rids *Drosophila* of microbial infections also defends the host against metazoan parasites. However, because of critical differences in their size and mode of entry, the combination of immune responses summoned in the two cases is different. While phagocytosis and systemic humoral responses (the latter originating from the fat body and in the gut) are the principal mechanisms of host defense against bacteria and fungi [2], [33], the development of parasitic wasp eggs is blocked primarily by encapsulation response [1], [2], [3], [4].

**Figure 9:**
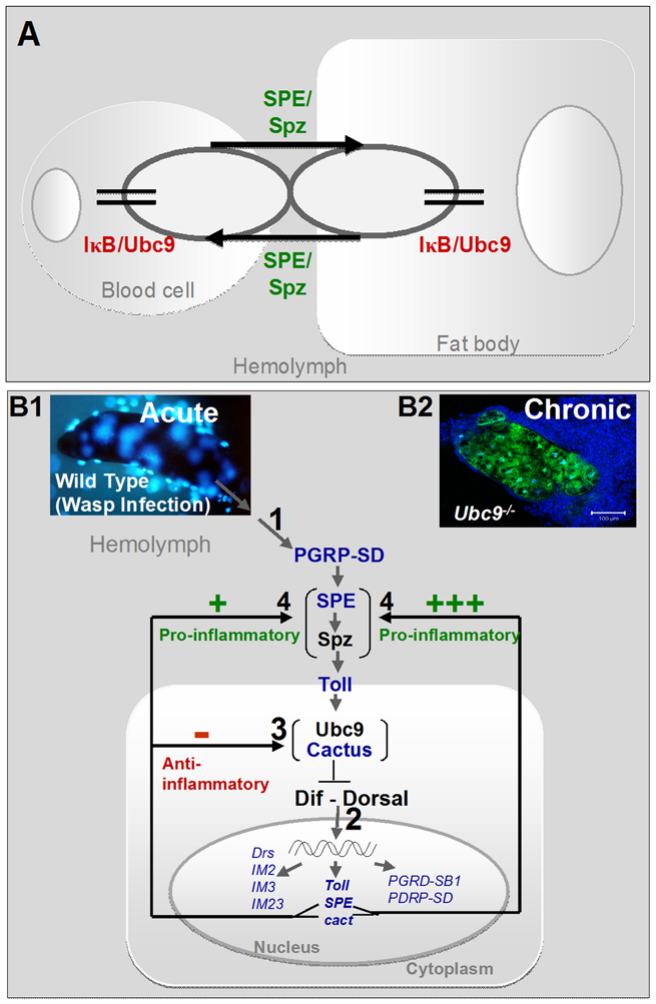
Regulatory immune circuit for host defense against wasp encapsulation. (A) A bi-directional cross-organ interaction involves a common regulatory circuit shown in detail below. Active Spätzle (SPE/Spz) acts cell non-autonomously to mediate this interaction. In each organ, sumoylation plays a regulatory role in restoring immune homeostasis. (B1) Acute infection by parasite activates the Toll-NF-κB cascade (step 1). Infection results in activation of, among others, pro- (*SPE*) and anti-inflammatory (*cactus*) targets (step 2). In a conserved feedback mechanism, Cactus protein is stabilized by Ubc9 (step 3). (Transcription of components in blue was identified to be activated in the microarray datasets; Table S1.) High SPE levels activate Spz in the extracellular compartment (step 4). SPE/Spz stimulates pro-inflammatory reactions via Toll (step 4). Sustained Cactus inhibition dampens the SPE/Spz cues and inflammation is resolved. (B2) In the absence of negative regulation via loss of Ubc9 (step 3), Cactus transcript levels are high, but protein levels remain low. Thus, signaling persists even in the absence of infection (step 1) because high levels of SPE/Spz build up (step 4). The amplification of this positive feedback step leads to chronic inflammation observed in *Ubc9^−^* mutants.

We present data that for the first time demonstrate the critical requirement of the humoral arm in both the activation and resolution of egg encapsulation (Figures 4 and 8). We show that the bi-directional interaction between the blood cells and the fat body occurs via cell non-autonomous effects of SPE/Spz, where these secreted proteins synthesized in one compartment can activate immune signaling in the other (Figure 9A). Recent reports corroborate a signaling role for Spz derived from blood cells in the expression of antimicrobial peptides from the larval fat body in response to microbes [34], [35]. Because activation/deactivation of both immune arms is accomplished via the IκB/Ubc9-dependent feedback loop that has both, cell autonomous and cell non-autonomous effects (Figure 9A, also see below), we propose that this shared mechanism allows efficient coordination between the immune organs and helps restore normal immune homeostasis within the infected host.

### Sumoylation balances activation and resolution of inflammation *in vivo*

The mechanism that coordinates the activation and resolution of both immune arms after parasite infection involves a balance between the positive (SPE) and negative (Cactus) components. Infection induces nuclear localization of Dorsal and Dif, and the transcription of both *SPE* (which resolves over time) and *cactus* (transcription levels off) (Figures 3 and S4). This Cactus-dependent regulation is essential for the downregulation of *SPE* transcription and the termination of the encapsulation response (Figure 9B). The negative feedback loop of Cactus in flies is similar to the one identified for IκBα in mammalian cells [36].

In *Ubc9* mutants, the stability of Cactus protein is compromised, and Toll signaling persists during the extended larval life. Accordingly, knockdown of Cactus in blood cells (*Hml>cactus^RNAi^*) promotes inflammation, aggregation and melanization (our unpublished results). We propose that loss of immune homeostasis leads to constitutive *SPE* expression and activation of Spätzle, which promotes the development of chronic inflammation (Figures 8 and 9B). Thus, sumoylation serves an anti-inflammatory function in the fly larva.

We have identified at least two distinct biological roles of sumoylation: first, an essential role in blood cells, where the post-translational modification curbs proliferation in the lymph gland in the absence of infection [17]. This conclusion is also strongly supported by restoration of normal hematopoietic complement in mutants expressing wild type Ubc9 only within a limited lymph gland population (Figure 2L, O, and data not shown). Second, sumoylation is essential to sustain significant, steady state levels of Cactus (Figure 8). In mammalian cells, sumoylation of IκBα protects it from antagonistic, ubiquitination-mediated degradation [21]. Our results are consistent with the mammalian model where Cactus sumoylation would be expected to modulate its half-life. We are testing this idea in ongoing experiments.

### Distinct roles for Spz in hematopoiesis and immunity

Cytokine activation and function are hallmarks of the normal inflammatory response in mammals. A key finding of our study is that active Spz serves a pro-inflammatory function in fly larvae. This first report of any pro-inflammatory molecule in the fly confirms that cytokines activate inflammation across phyla. As with mammalian cytokines that act as immuno-stimulants, Spz is expressed, and is therefore likely to activate the blood cells surrounding the parasite capsule (Figure 5). Active Spz promotes blood cell division, migration and infiltration (Figure 7) much like high levels of Dorsal and Dif, suggesting that the cell biological changes triggered by SPE/Spz are mediated by target genes of Dorsal and Dif. It is intriguing that the integrity of the basement membrane (as visualized by Collagen IV expression pattern) appears to be important for orchestrating blood cells to the site of “diseased self” (here, mutant fat body) in a manner that may be similar to recognition of the non-self parasitic egg, underscoring the parallel roles of basement membrane proteins in the origin and development of inflammation in both flies and mammals [10].

Although excessive (active) Spz is proinflammatory, its loss leads to reduction in the hematopoietic complement. For example mutants lacking spz (s*pz^rm7^/spz^rm7^*) exhibit a 40% reduction in circulating blood cell concentration and these animals do not encapsulate wasp eggs as efficiently as their heterozygous siblings [7]. These observations suggest that active Spz's normal proliferative/pro-survival functions, required for maintaining the normal hematopoietic complement, are fundamentally linked to its immune function for the activation and recruitment of blood cells to target sites. Thus, the autocrine and paracrine hematopoietic and inflammatory effects of Spz are amplified in the presence of hyperactive Toll receptor, excessive Dorsal/Dif, or the loss of Cactus/Ubc9 inhibition, resulting in production of hematopoietic tumors [18], [29]. It is possible that mutations in other, unrelated, genes that yield similar inflammatory tumors arise due to the loss of Toll-NF-κB dependent immune homeostasis.

Our results highlight the central role of the Dorsal/Dif proteins not only in immune activation, but also in the resolution of these responses. Recent proteomic studies [37] have confirmed that Dorsal is a *bona fide* SUMO target and its transcriptional activity is affected by sumoylation [38]. Dorsal and Dif exhibit genetic redundancy in both the humoral and cellular responses [17], [18], [39]. It is possible that this redundancy ensures that immune reactions against microbes and parasites are efficiently resolved to allow proper host development.

### Implications

In nature, parasitic wasps are continually evolving to evade or suppress the immune responses of their hosts. To this end, they secrete factors or produce protein complexes with specific molecular activities to block encapsulation. Our studies provide the biological context in which the effects of virulence factors produced by pathogens and parasites on primordial immune pathways can be more clearly interpreted. The molecular identity of wasp factors which actively suppress humoral and cellular responses (e.g., those in *L. heterotoma* [7]) remains largely unknown [4]. Such virulence factors are likely to be “anti-inflammatory” as they clearly interfere with host physiology [4], [7] that ultimately disrupts the central regulatory immune circuit defined in our studies.

Encapsulation reactions of non-self (wasp egg) or diseased self tissues (fat body) of the kind in the *Drosophila* larva are not only reported in other insects [40], but the reaction is likely to be similar to mammalian granulomas, which are characterized by different forms of localized nodular inflammation [11]. Furthermore, the phenotypes arising from persistent signaling in mutants recapitulate the key features of mammalian inflammation: i.e., reliance on conserved signaling mechanism, the requirement for cytokines, and sensitivity to aspirin (our unpublished results). Our studies also reveal a clear link between innate immunity and the development and progression of hematopoietic cancer in flies, as has been hypothesized from work in mammalian systems [9], [41], [42], [43]. In the past, genetic approaches in *Drosophila* have served well to dissect signaling mechanisms governing developmental processes in animals. The fly model with hallmarks of acute and chronic mammalian inflammatory responses will provide deep insights into signaling networks and feedback regulatory mechanisms in human infections and disease. It can also be used to test the potency and mechanism of action of pesticides, anti-inflammatory and anti-cancer agents *in vivo*.

## Materials and Methods

### Microarray data analysis

Published [5], [8] microarray datasets were formatted and compared to compile an initial list of putative inflammation genes in *Drosophila*. First, from the Wertheim dataset, 162 probe sets that were differentially regulated after *A. tabida* infection, 53 (classified as defense and/or immune related) were selected based on gene expression profiles. In the Schlenke dataset, 589 probe sets were differentially regulated after *L. boulardi* infection, of which 95 (classified as defense related) were selected, also based on gene expression profiles. We manually selected only those probe sets with acute-phase kinetics whose expression profile showed strong activation minutes to hours after infection, but followed a downward trend 12 hours after infection. This exercise resulted in 81 putative inflammation genes (40 genes from the Wertheim study and 51 genes from the Schlenke study, with 10 genes common to both studies).

### Drosophila stocks

UAS lines: *UAS*-*Ubc9^WT^* [17], *UAS-Ubc9^RNAi^* (TRiP Valium 1), *UAS-Aos1^RNAi^* (Vienna Stock Center) and *UAS-Uba2^RNAi^* (Valium 10 stock), *UAS-EGFP-Dorsal* and *UAS-CFP-Dif* (from T. Ip; [44]); *UAS-SPE-Act* (amino acid 135–400 from B. Lemaitre; [14]); *UAS-Spz* (From B. Lemaitre); *UAS-Spz-Myc*, *UAS-Spz-V5* (from T. Ip; [45]), *C564-Gal, UAS-SPE^RNAi^* (from B. Lemaitre, *C564* is expressed in the fat body and the hemocytes; [30]). Full-length Spätzle is 326 amino acids long and the above transgenic lines contain the entire coding region of the protein. When overexpressed in immune tissues, all three transgenic lines produced the same biological effects.

Gal4 lines: Gal4 lines, their sources and expression patterns are as follows: *SerpentHemo-Gal4* (*srp* is expressed in all blood cells; [46]; stock obtained from Dr. M. Meister), *Cg-Gal4* (*Cg* is expressed in fat body, circulating hemocytes and some cells of the lymph gland; [24]; Bloomington line 7011), *76B-Gal4* (*76B* is expressed in some cells of the medullary zone of the lymph gland, ring gland, genital disc, salivary gland; [26]; obtained from Dr. D. Harrison; *Lsp2-Gal4* (*Lsp* is expressed in the fat body; [27] Dr. Hao Li), *Hml-Gal4* (*Hml* is expressed in circulating hemocytes and some cells in the lymph gland; [28]; obtained from Dr. S. Bhattacharya). The *y w pUAST-EGFP-Dorsal* line was recombined into *Srp-Gal4* background and was balanced with *Basc* to generate *y w Srp-Gal4 UAS GFP-dl/Basc* stock.

*Ubc9* stocks and recombinants: *Drosophila Ubc9* is synonymous to *lesswright* (*lwr*). Mutant *Ubc9^4-3^/Ubc9^5^* animals are developmentally-delayed and most mutants die by day 10 as larvae.

*Ubc9* stocks *y w*; *Ubc9^4-3^ FRT40A/CyO y^+^, y w; Ubc9^5^ FRT40A/CyO y^+^, y w; Drs-GFP, Ubc9^4-3^/CyO y^+^, y w; Drs-GFP Ubc9^5^/CyO y^+^* are described previously [17]. The *Ubc9* alleles were recombined or crossed into the Gal4 or UAS backgrounds to produce: *y w; Ubc9^5^, Cg-Gal4/CyO y^+^, y w, UAS-CFP-Dif; Ubc9^4-3^/CyO y^+^, y w; Ubc9^4-3^, UAS-EGFP-dl/CyO y^+^*.

For rescue, a *76B-Gal4* insert [26] was recombined with the *Ubc9^5^* mutation on a chromosome carrying *Drs-GFP* transgene [47]. Males from this stock were crossed with *UAS- Ubc9^WT^; Drs-GFP, Ubc9^4-3^/CyO y* ^+^ females [17]. Mutants carrying *76B>Ubc9^WT^* were compared to mutants without either *UAS- Ubc9^WT^* or *76B-Gal4* transgene.

*Dif, dorsal, cactus* stocks: Stocks lacking Dif and Dorsal, *y w; Df(2L)J4/CyO y^+^* and *y w; b Df(2L)119/CyO y^+^* and *cactus* stocks *yw*; *Drs-GFP, Ubc9^4-3^ cact^E10^/CyO y^+^, y w; Drs-GFP Ubc9^5^ cact^E10^/CyO y^+^* are described previously [17]. P{FZ}-*cact^255^*/*CyO* is a *P-lacZ* insertion allele [31]. *hsp83-lacZ* line 25 has high levels of ubiquitous β-galactosidase expression [48] and was used to compare β-galactosidase expression in the *cact^255^* stock.

*spätzle* mutant: The *Ubc9* alleles were crossed into the original line *rut h s tri roe p^p^ e spz^rm7^/TM3 Sb Tb* [7] to produce double mutant stocks of the genotype *w; Ubc9^5^/CyO actin GFP; spz^rm7^/TM6 Tb* and *w; Ubc9^4-3^/CyO actin GFP; spz^rm7^/TM6 Tb*. Double mutants, *Ubc9^4-3^/Ubc9^5^; spz^rm7^/spz^rm7^* or *Ubc9^4-3^ cact^E10^/Ubc9^5^ cact^E10^* are lethal during late larval stages.

### Wasp infections

Standard protocol [3] was used for rearing wasps on the *y w* fly strain. Wasp stocks used were *L. victoriae* [49] and *L. boulardi* strain 17 [5]. Infections were performed on 3-day-old larvae (after 72 hrs of egg-laying). For real time PCR experiment, 50 early third-instar *y w* larvae were exposed to 10 *L. boulardi*-17 females for 2 hours. To assess *Drs-GFP* expression, third-instar *y w; P{w^+^ Drs-GFP}* larvae were exposed for 24 hours. As a positive control, larvae were challenged by poking with a sterile glass needle. In all cases, success of infection was confirmed by dissection of parasite eggs from the host. To examine the role of SPE in wasp encapsulation, second instar *c564>SPE^RNAi^* larvae were superinfected with *L. victoriae*. Third instar larvae were dissected to document the number of wasp larvae, melanized aggregates and capsules per host larva. The experiment was done twice and more than 120 animals; in each attempt more than 60 infected hosts were examined.

### RNA collection and real time-PCR

Fifty developmentally-synchronized animals of the appropriate genotypes (6 or 8 days after egg lay) or *y w* larvae (2, 6, 12 or 24 hours after infection) were collected for RNA extraction (Trizol method, GibcoBRL, Invitrogen, Carlsbad, CA). RNA was quantified by a Smartspec 3000 (Bio-Rad Laboratories, Hercules, CA). 2 µg of total RNA served as template for cDNA synthesis (Protoscript first strand synthesis kit; New England BioLabs, Ipswich, MA). Real time PCR was performed running the standard two-step PCR program: 0.5 µl of the cDNA sample was mixed with iQ SYBR Green Supermix (Bio-Rad Laboratories) and primers to set up a 25-µl reaction mix. Transcript levels detected were normalized to *rp49* mRNA values. Primers used:

***Drosomycin***: Forward primer (5′) ATC CTG AAG TGC TGG TGC GAA GGA (3′); Reverse primer (5′) ACG TTC ATG CTA ATT GCT CAT GG (3′)

***spätzl*** *e*: Forward primer (5′) GGA GCG GAT CAA CCC TGT G (3′); Reverse primer (5′) TTG GAT TAT AGC TCT GCG GAA AG (3′)

***SPE***: Forward primer (5′) CTT TTC GCT GAT CGC ATT TT (3′); Reverse primer (5′) CAC CGG ATT TGT CCA GTT CT (3′)

***cactus***: Forward primer (5′) CTG CTC AAC ATC CAG AAC GA (3′); Reverse primer (5′) GCC GAA CTT CTC TGT CAA GG (3′)

***hopscotch***: Forward primer (5′) AAT AAT CCA CGG CTC GTC AG (3′); Reverse primer (5′) ACG CTT GCT TTT CGC ATA GT (3′)

***Irc***: Forward primer (5′) TGG CTG AAA AAT CCG AGT TC (3′); Reverse primer (5′) GTC CAA CGC CGT TTC TAC AT (3′)

***rp49***: Forward primer (5′) GAC GCT TCA AGG GAC AGT ATC TG (3′); Reverse primer (5′) AAA CGC GGT TCT GCA TGA G (3′)

### Infiltration index, tumor penetrance, and *Drs-GFP* expression

Fat body from control, mutant and experimental animals was dissected, fixed, stained with Hoechst and TRITC labeled phalloidin, and then mounted with 50% glycerol in PBS. The number of single blood cells adhering to the top and bottom of the fat body were scored using a Zeiss Axioscope fluorescence microscope. Aggregates attached to the fat body were not scored. Whole larvae were scored for melanized microtumors through the cuticle. Transcriptional activity of the *Drs-GFP* promoter was analyzed in whole larvae under a Leica stereomicroscope equipped with GFP-compatible fluorescence. Student *t*-test was performed using SAS (SAS, Inc., Cary, NC) to determine statistical significance.

### Immunostaining, microscopy, data collection and analysis

Developmentally synchronized larvae were dissected to collect fat bodies or blood cells. Samples were fixed with 4% paraformaldehyde, prepared in 2% sucrose in PBS at pH 7.6. Fixed samples were blocked in 3% bovine serum albumin, followed by an overnight incubation with primary antibody (mouse monoclonal anti-dorsal (7A4, 1∶10; [50]), mouse monoclonal anti-Cactus (3H12, 1∶20; [50]), mouse monoclonal anti-integrin-β PS (1∶50, [51]) all obtained from Developmental Studies Hybridoma Bank, University of Iowa). Integrin-β PS is expressed in blood cells [52]. Rabbit polyclonal anti-Collagen IV (1∶500, [25], gift of Dr. L. Fessler), rabbit polyclonal anti-Spätzle (1∶100, [14], gift of Dr. C. Hashimoto), plasmatocyte-specific monoclonal mouse anti-Nimrod C (1∶10, [53], gift of Dr. I. Ando), rabbit polyclonal anti-phospho histone H3 (1∶200, Upstate Cell Signaling Solutions), and rabbit anti-β-galactosidase (1∶1, MP Biomedicals, LLC). Washed samples were incubated for 3 hrs at room temperature with commercially available secondary antibody (TRITC/FITC-conjugated donkey anti-rabbit, 1∶50 or TRITC/FITC-conjugated donkey anti-mouse, 1∶50, Jackson Immuno Research Laboratories). After three washes, samples were counterstained with nuclear dye Hoechst 33258 (Invitrogen Molecular Probes, Eugene, OR) and/or TRITC (Tetramethyl Rhodamine Iso-Thiocyanate)-labeled phalloidin (0.5 µg/ml, Invitrogen Molecular Probes, Eugene, OR), and then mounted in 50% glycerol in PBS or Vectashield (Vector Laboratories, Inc., Burlingame, CA). To assess Dorsal and Dif subcellular localization, CFP-Dif and GFP-Dorsal, driven by *Cg-Gal4*, were monitored in fat body of 6 or 7 day-old third instar larvae, after a six hour egg-lay from animals reared at 28°C. Images were obtained using a Zeiss LSM510 confocal microscope or Zeiss Axioplan 2 equipped with fluorescence optics. Images for control and experimental samples were taken at identical settings. Images were processed identically for quantitative analysis of signals.

Quantification of the fluorescence signal after anti-Cactus antibody staining was done in Adobe Photoshop CS3 (Adobe Systems Inc., San Jose, CA). At least 300 cells from 9 larvae were analyzed for each genotype. A one-way ANOVA analysis was performed in SAS (SAS Inc., Cary, NC). We partitioned and contrasted the different genotypes to identify significant pair-wise differences by comparing *p*-values on a PDIFF matrix and applying sequential bon Ferroni corrections to adjust for accumulated error.

### Dorsal and Dif/Relish binding sites

We queried both Dif/Relish (GGGAWTCMC; [54]) and Dorsal binding sites GGGWDWWWCCM or GGGWWWWCCM; [55]) consensus sequences in the fly genome using Fly Enhancer (opengenomics.org). To yield all possible binding sites for these consensus sequences, we queried one cluster per 400 bp frame. The Dif/Relish consensus sequence query obtained close to 3,000 predicted target binding sites, but none mapped to either the *cactus* or *SPE* regions. Search for the Dorsal consensus sequences yielded over 10,000 predicted target binding sites, with multiple hits mapping to either the *cactus* or *SPE* regions (Figure S2).

Approximately 6 kb of *SPE* (∼5 kb flanking) and 17 kb of *cact* (∼2 kb flanking) genomic sequences containing coding and flanking sequences were retrieved in FASTA file format from Fly Base Gbrowser [56]. Known exons and untranslated regions were annotated on the *cactus* and *SPE* genomic sequences using NCBI Sequence Viewer (http://www.ncbi.nlm.nih.gov). To confirm *dorsal* consensus sequence predictions, we processed consensus sequences through GeneQuest (DNAStar, Inc., Madison, WI) keeping the threshold at 100% with bi-directionality match.

## Supporting Information

Table S1 Inflammation genes in *Drosophila*. A list of 81 acute phase genes derived from genome-wide wasp infection studies of *D. melanogaster*. Infecting wasps were *L. boulardi*-17 [5] or *A. tabida* [8]. While infection by either wasp activates the transcription of *Drosophila* genes, they have starkly different parasitization strategies. *A. tabida* lacks virulence factors and its infection provokes a strong encapsulation response in the host [8]. *L. boulardi*-17 does not trigger strong encapsulation in *D. melanogaster* larvae because of immune evasion and the presence of virulence factors [5]. Genes are grouped in the Table based on their known or predicted functions in immune physiology. Genes within a cluster share the same expression profile. Forty genes in Table S1 and Figure S1A from the *A. tabida* study belong to gene clusters 1, 2, 4, 11, 12. These clusters were created by Wertheim *et al.* (2005), who performed the genome-wide analysis over a 72 h period [8]. Based on acute-phase kinetics of individual genes, we created clusters A-C for 51 genes in Table S1 and Figure S1B from the Schlenke study [5] which was carried out over a 24 h time period.
(0.03 MB XLS)

Figure S1 Activation profiles of *Drosophila* inflammation genes in response to wasp parasitization. Activation profiles are derived from two previous studies: Wertheim *et al*., 2005 [8] for *A. tabida* infection (A) and Schlenke *et al*., 2007 [5] for *L. boulardi* infection (B). Genes within a cluster in each study follow a similar up- and down-regulation profile as a function of time (also see Methods and legend to Table S1). (A) Microarray profiles of genes in *A. tabida*-infected hosts (0-72 h time period) were grouped in several gene clusters by the authors [8]. Of these clusters 1, 2, 4, 11 and 12 are shown and they include 40 genes. Genes in all these clusters exhibit acute-phase profile, although their exact course differs as shown. The identity of the 40 genes within each cluster is shown in Table S1. (B) Gene expression profiles of *L. boulardi* 17 infected hosts (Schlenke, 3 time points [5]) with acute-phase profile were grouped in 3 clusters (A, B, C) based on overall trends of expression over the 24-hour period. The identities of all 51 genes within these three clusters are shown in Table S1.
(10.18 MB TIF)

Figure S2 Putative binding sites for transcription factor Dorsal in *SPE* and *cactus* genes. Location of Dorsal-binding sites relative to the transcription start sites of the *SPE* (A) and *cactus* (B) loci. Directionality (forward or reverse), the target binding sequence, and the consensus sequence in each gene are shown in the respective Tables below the schematics.
(6.75 MB TIF)

Figure S3 Spätzle expression in larval blood cells and fat body. (A-C) Plasmatocytes from heterozygous *spz^+/-^* animals (A, B) either not treated (A) or treated (B, B') with anti-Spz antibody (red). Plasmatocytes from homozygous *spz^-^* animals stained with anti-Spz antibody (C, C'). (D-F') Fat body cells from *Ubc9^-^* mutants treated with secondary but not primary antibody (D). Heterozygous fat body (E, E') shows lower Spätzle levels than *Ubc9^-^* fat body (F, F'). (A-F) Cells were counterstained with Hoechst (blue). Panels B', C', E' and F' show the expression of Spz alone.
(7.47 MB TIF)

Figure S4 Regulation of *cactus* transcription and Cactus protein levels after parasite infection. (A-C) Transcriptional activation of the *cactus^255^-lacZ* in the larval fat body after wasp infection. Samples were stained with anti-β-galactosidase antibody (green). Fat body from uninfected (A), *L. victoriae*-infected (B), or *L. boulardi*-infected (C) animal (12 h post-infection). (D-H) Transcriptional activation of the *cactus^255^-lacZ* in the blood cells after wasp infection. Blood cells from wild type larvae without the *lacZ* transgene (D), *hsp83-lacZ* (constitutive, control) (E), uninfected *cact^255^/+* (F), *L. victoriae-*infected (G, G'; arrows in latter point to lamellocytes), or *L. boulardi-*infected (H) larvae. Cells from infected animals were recovered 24 hours after infection. Pixel intensity quantification of the β-galactosidase signal reveals that *cact* transcription increases 17-fold after *L. victoriae* (panel G) and 21-fold after *L. boulardi* (panel H) infection. As a reference, the signal intensity in *hsp83-lacZ* cells (panel E) is roughly 24-fold relative to the average signal in cells from uninfected animals expressing the *lacZ* transgene (panel F) (data not shown). (I-L) Larval fat body cells stained with anti-Cactus antibody (red). Fat body cells treated with secondary but not primary antibody (I). Fat body from an uninfected animal (J). Fat body from *L. victoriae-*infected (K), or *L. boulardi-*infected (L) animals, 12 h post-infection. (A-L) Cells were counterstained with Hoechst (blue).
(7.69 MB TIF)
