# Supplementary figures and images for: Role for Sumoylation in Systemic Inflammation and Immune Homeostasis in Drosophila Larvae
Source: PLoS Pathog. 2010 Dec 23;6(12):e1001234. doi: 10.1371/journal.ppat.1001234 (PMC3009591; doi:10.1371/journal.ppat.1001234)

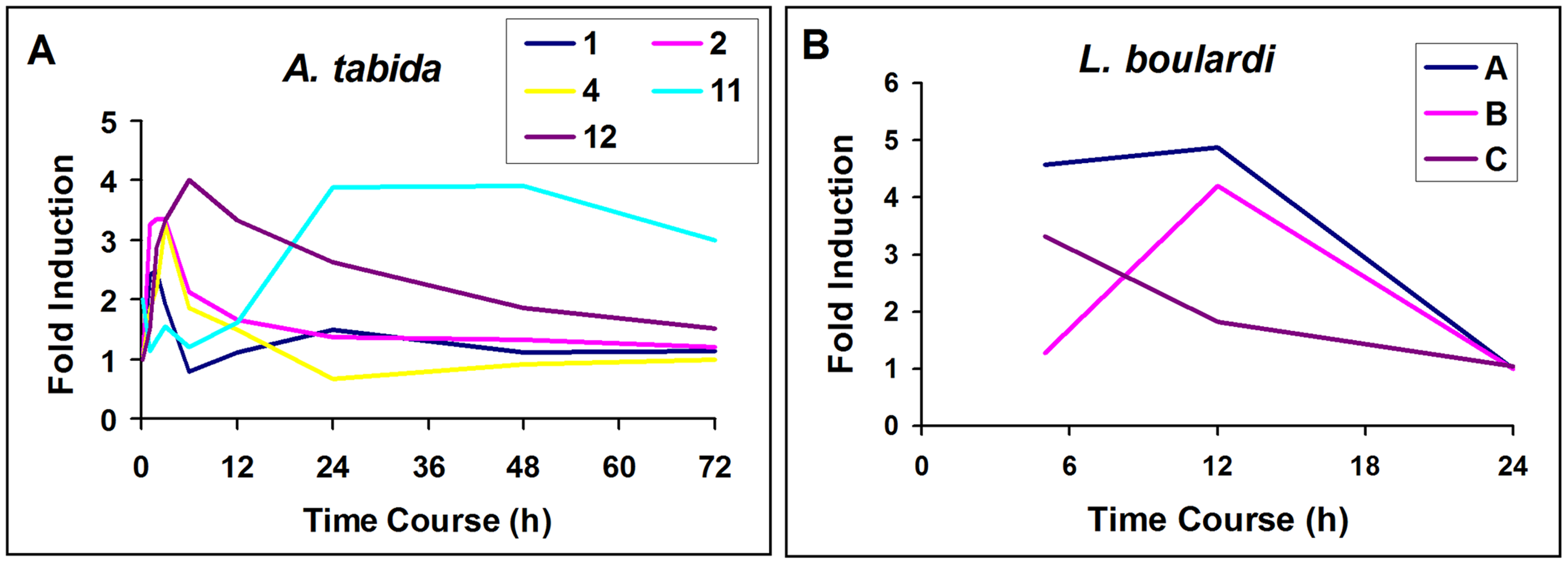

Supplement: Figure S1 — Activation profiles of Drosophila inflammation genes in response to wasp parasitization. Activation profiles are derived from two previous studies: Wertheim et al., 2005 [8] for A. tabida infection (A) and Schlenke et al., 2007 [5] for L. boulardi infection (B). Genes within a cluster in each study follow a similar up- and down-regulation profile as a function of time (also see Methods and legend to Table S1). (A) Microarray profiles of genes in A. tabida-infected hosts (0-72 h time period) were grouped in several gene clusters by the authors [8]. Of these clusters 1, 2, 4, 11 and 12 are shown and they include 40 genes. Genes in all these clusters exhibit acute-phase profile, although their exact course differs as shown. The identity of the 40 genes within each cluster is shown in Table S1. (B) Gene expression profiles of L. boulardi 17 infected hosts (Schlenke, 3 time points [5]) with acute-phase profile were grouped in 3 clusters (A, B, C) based on overall trends of expression over the 24-hour period. The identities of all 51 genes within these three clusters are shown in Table S1. (10.18 MB TIF) [file ppat.1001234.s002.tif]

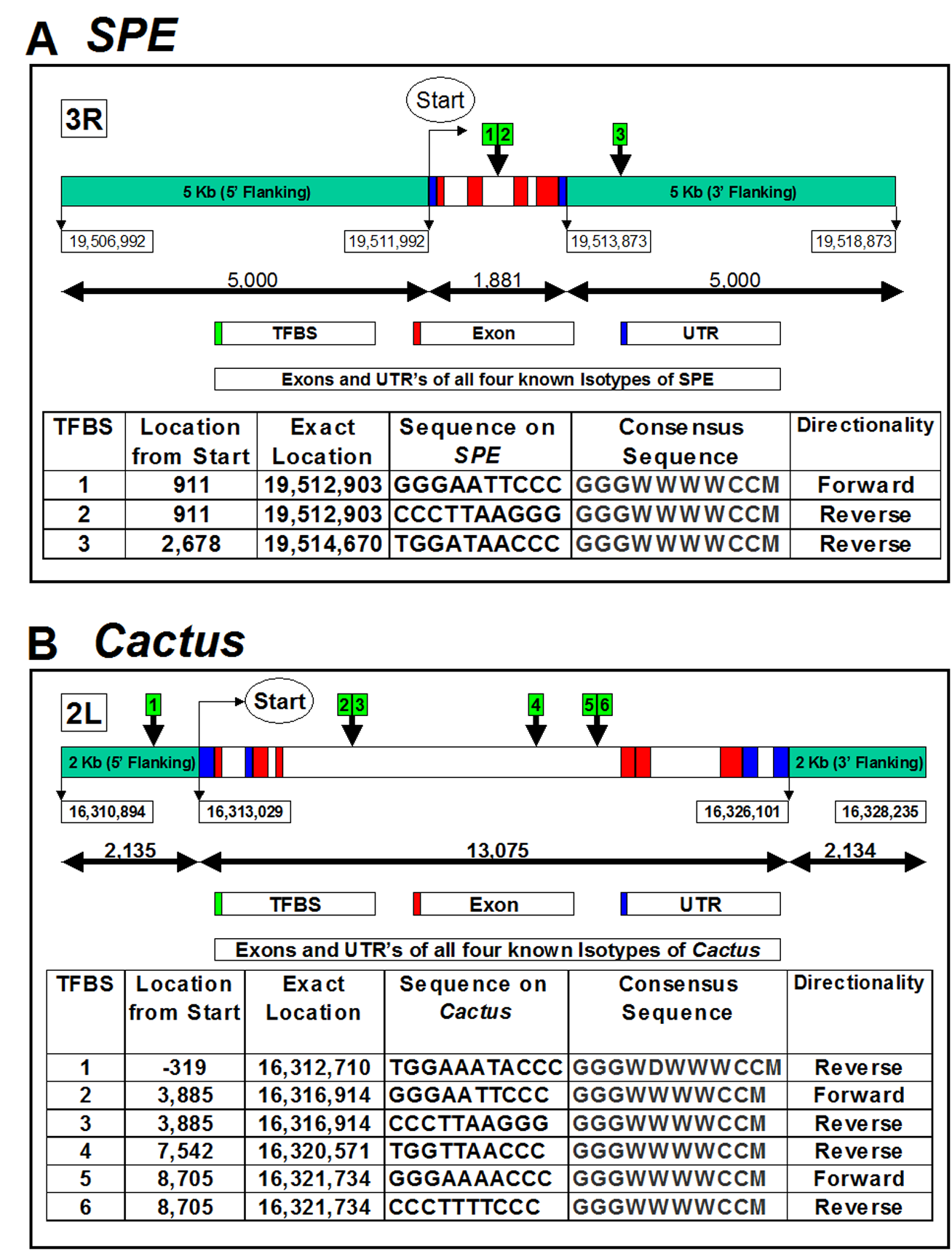

Supplement: Figure S2 — Putative binding sites for transcription factor Dorsal in SPE and cactus genes. Location of Dorsal-binding sites relative to the transcription start sites of the SPE (A) and cactus (B) loci. Directionality (forward or reverse), the target binding sequence, and the consensus sequence in each gene are shown in the respective Tables below the schematics. (6.75 MB TIF) [file ppat.1001234.s003.tif]

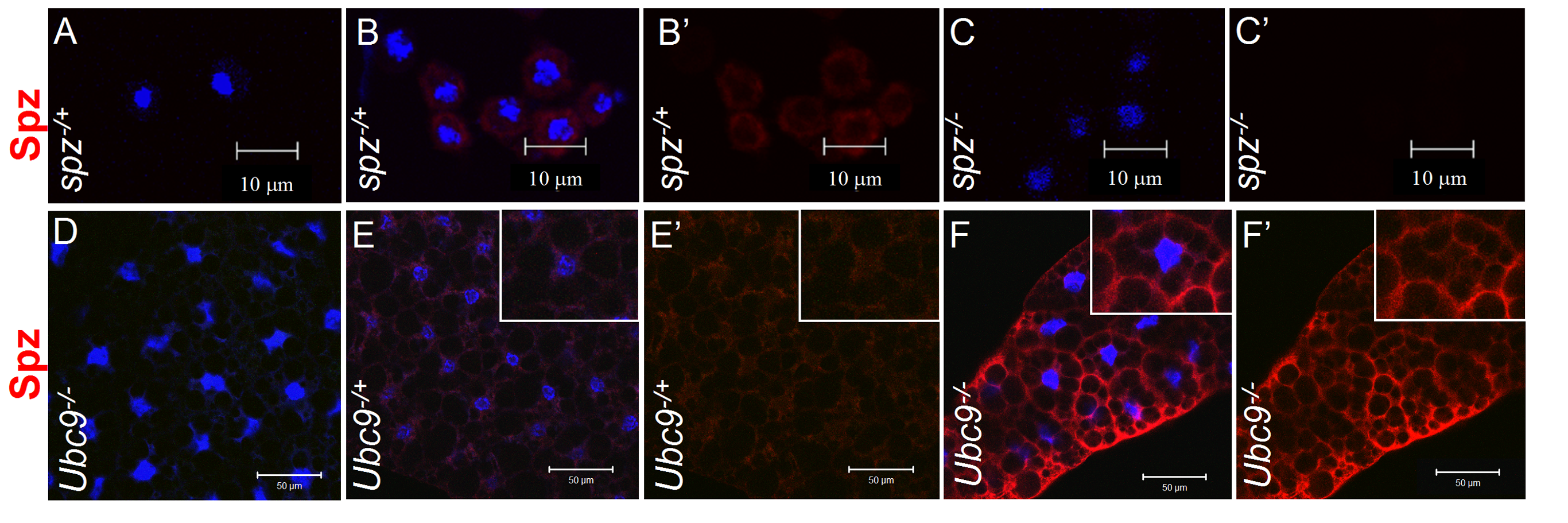

Supplement: Figure S3 — Spätzle expression in larval blood cells and fat body. (A-C) Plasmatocytes from heterozygous spz+/- animals (A, B) either not treated (A) or treated (B, B') with anti-Spz antibody (red). Plasmatocytes from homozygous spz- animals stained with anti-Spz antibody (C, C'). (D-F') Fat body cells from Ubc9- mutants treated with secondary but not primary antibody (D). Heterozygous fat body (E, E') shows lower Spätzle levels than Ubc9- fat body (F, F'). (A-F) Cells were counterstained with Hoechst (blue). Panels B', C', E' and F' show the expression of Spz alone. (7.47 MB TIF) [file ppat.1001234.s004.tif]

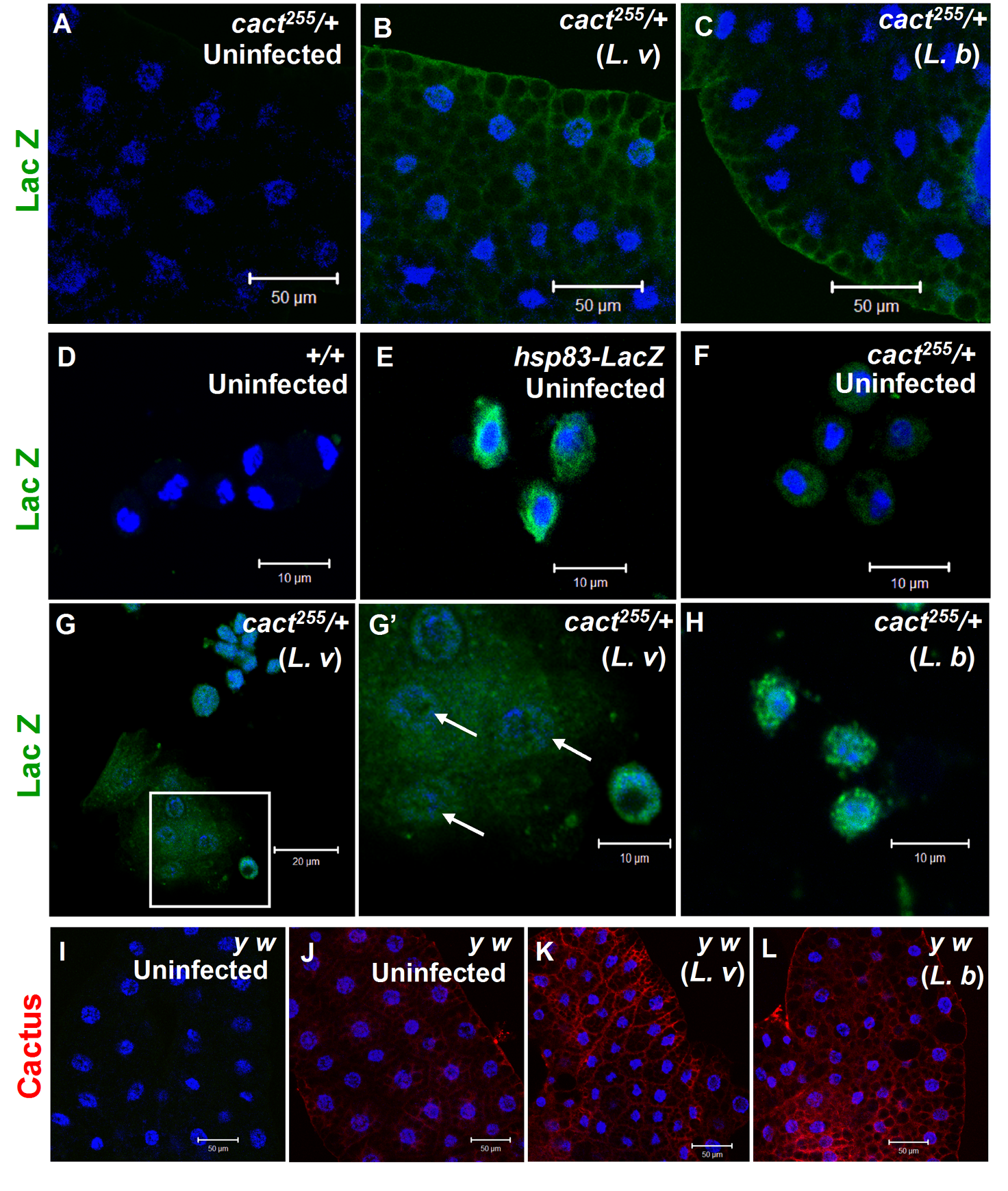

Supplement: Figure S4 — Regulation of cactus transcription and Cactus protein levels after parasite infection. (A-C) Transcriptional activation of the cactus255-lacZ in the larval fat body after wasp infection. Samples were stained with anti-β-galactosidase antibody (green). Fat body from uninfected (A), L. victoriae-infected (B), or L. boulardi-infected (C) animal (12 h post-infection). (D-H) Transcriptional activation of the cactus255-lacZ in the blood cells after wasp infection. Blood cells from wild type larvae without the lacZ transgene (D), hsp83-lacZ (constitutive, control) (E), uninfected cact255/+ (F), L. victoriae-infected (G, G'; arrows in latter point to lamellocytes), or L. boulardi-infected (H) larvae. Cells from infected animals were recovered 24 hours after infection. Pixel intensity quantification of the β-galactosidase signal reveals that cact transcription increases 17-fold after L. victoriae (panel G) and 21-fold after L. boulardi (panel H) infection. As a reference, the signal intensity in hsp83-lacZ cells (panel E) is roughly 24-fold relative to the average signal in cells from uninfected animals expressing the lacZ transgene (panel F) (data not shown). (I-L) Larval fat body cells stained with anti-Cactus antibody (red). Fat body cells treated with secondary but not primary antibody (I). Fat body from an uninfected animal (J). Fat body from L. victoriae-infected (K), or L. boulardi-infected (L) animals, 12 h post-infection. (A-L) Cells were counterstained with Hoechst (blue). (7.69 MB TIF) [file ppat.1001234.s005.tif]
